# Dataset on nicotine-free, nontransgenic tobacco (*Nicotiana tabacum*l.) edited by CRISPR-Cas9

**DOI:** 10.1016/j.dib.2019.104395

**Published:** 2019-08-23

**Authors:** Julia Schachtsiek, Felix Stehle

**Affiliations:** Laboratory of Technical Biochemistry, Department of Biochemical and Chemical Engineering, TU Dortmund University, Dortmund, Germany

**Keywords:** CRISPR-Cas9, Nicotine-free, Gene editing, Nontransgenic, *Nicotiana tabacum*, Non-addictive

## Abstract

This dataset in brief is related to the research letter entitled “Nicotine-free, nontransgenic tobacco (*Nicotiana tabacum*l.) edited by CRISPR-Cas9” [1]. Cured tobacco products with a significantly reduced nicotine content helps people to overcome their nicotine addiction. Here we summarize additional data and method descriptions of the generation process of a nicotine-free, nontransgenic tobacco plant. This included the cloning, transformation and regeneration of transgenic tobacco plants, followed by the analysis of the nicotine content and genomic modifications caused by CRISPR-Cas9 mediated gene editing. Subsequently, nicotine-free plants were screened for loss of T-DNA cassette, i.e. nontransgenity. Finally, a metabolic footprint was recorded by ^1^H NMR analysis.

**Table:** Specifications Table

Subject	Plant Science
Specific subject area	Plant Biotechnology, Plant genome editing
Type of data	Table and Figures
How data were acquired	Gibson cloning, Agrobacterium-mediated transformation, plant regeneration in tissue culture, PCR, sanger sequencing, gas chromatography, gas chromatography mass spectrometry, ^1^H NMR
Data format	Analyzed
Parameters for data collection	Tobacco plants were cultivated on hydro culture at 25 °C under long day conditions (18 h light/6 h dark) with a light intensity of 110 μM m^−2^ s^−1^.
Description of data collection	i) Nicotine content was analyzed by GC and GC-MS by extracting dried, grounded leaf material ii) Sequence data were obtained by Sanger sequencing of PCR products iii) metabolomic alterations were analyzed by ^1^H-NMR
Data source location	Dortmund, Germany
Data accessibility	With the article
Related research article	Julia Schachtsiek, Felix Stehle Nicotine-free, nontransgenic tobacco (*Nicotiana tabacum*l.) edited by CRISPR-Cas9 Plant Biotechnology Journal https://doi.org/10.1111/pbi.13193

**Table:** 

**Value of the Data** • First nicotine-free, nontransgenic tobacco plant that enables the production of non-addictive cured tobacco. • Nicotine-free smoking products can support people to overcome nicotine addiction. • This technology can be transferred to commercially used *N. tabacum* varieties as well as *N. benthamiana* to improve the biotechnological production properties. • First technology that eliminates the nicotine content while all other metabolites are not affected.

## 1 Data

**Fig. 1:**
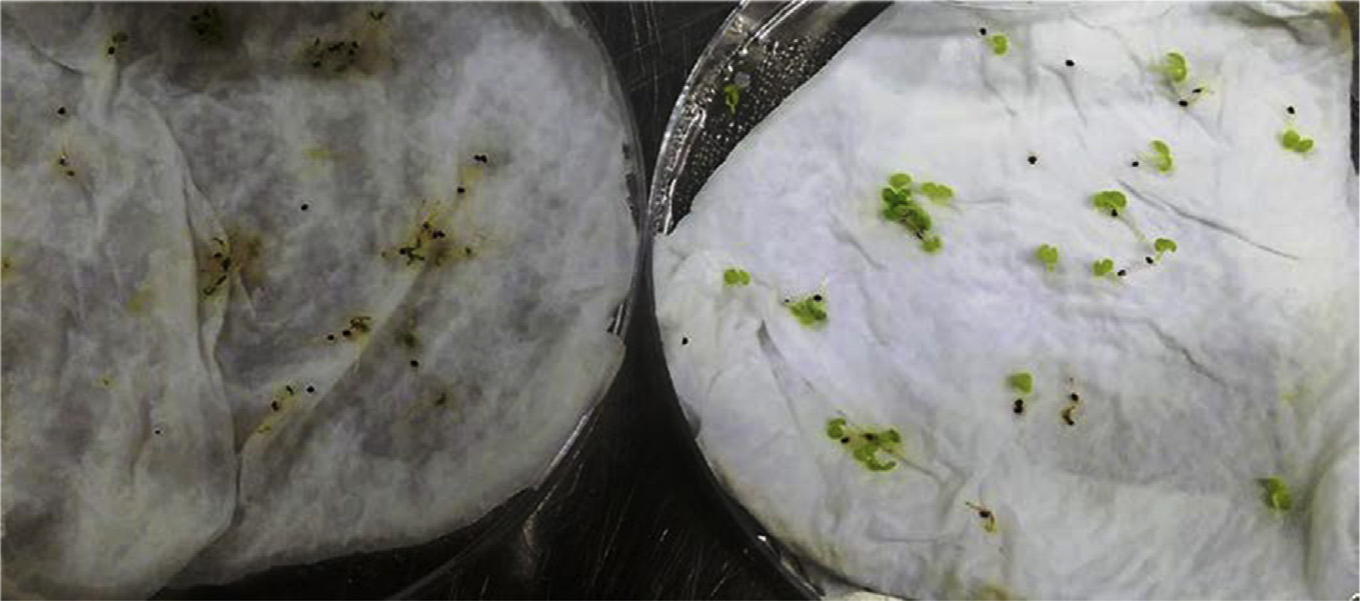
Selection for positive transformants in T_1_-generation: Seeds from T_0_ – plants were germinated on tissue paper and sprayed with a 100 mg L^−1^ PPT solution three times every two days. Seedlings of the wild type (left) died, seedlings from a transformed and regenerated plant (right) survived.

**Fig. 2:**
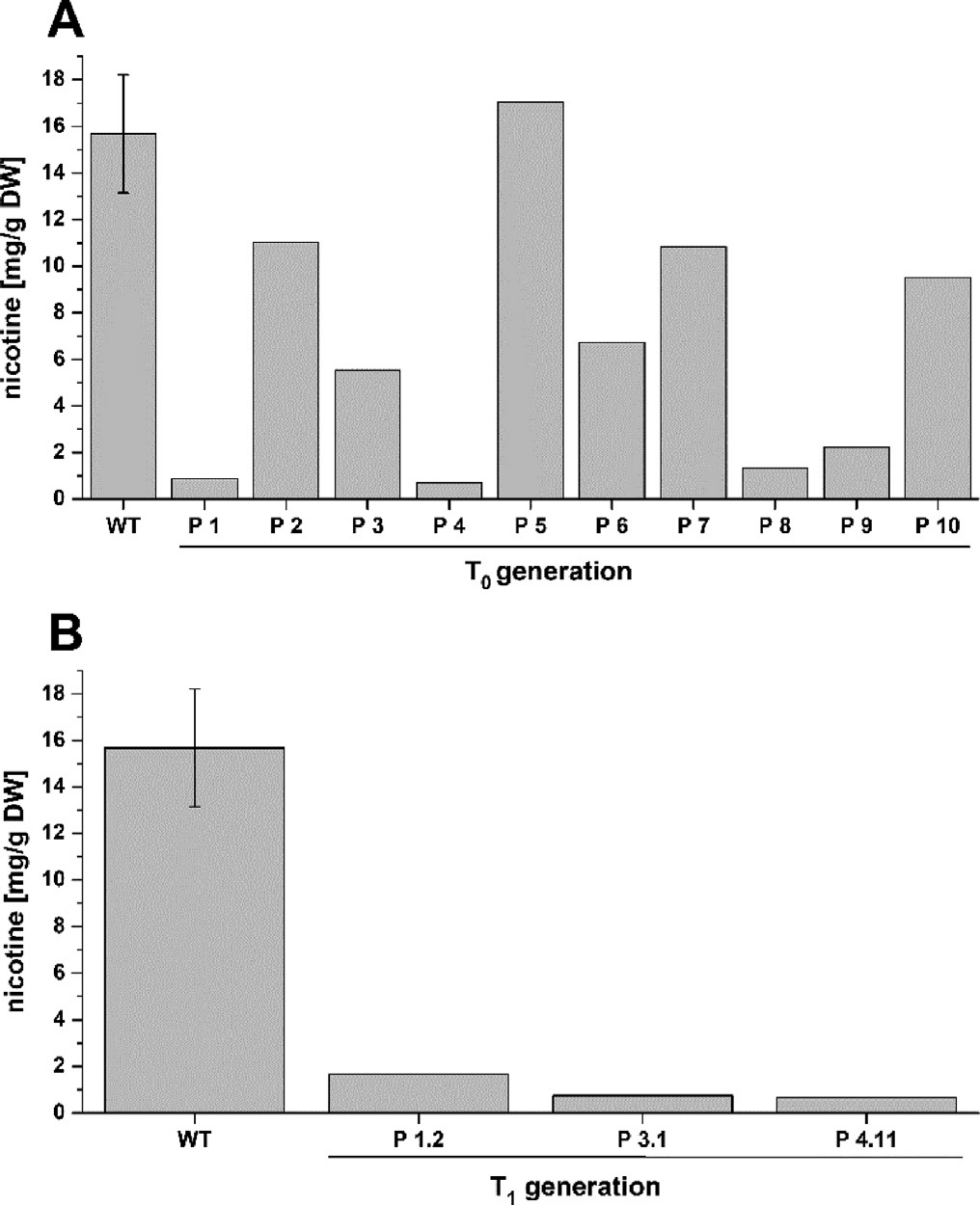
Amount of nicotine in wild type, T_0_ and T_1_ plants: (A) the amount of nicotine was measured with GC-FID of 200 mg dried and grounded leaf material of plants extracted with MTBE. Amount of nicotine was calculated as mg per gram dry weight (DW). (B) amount of nicotine in extracts of T_1_ plants.

**Fig. 3:**
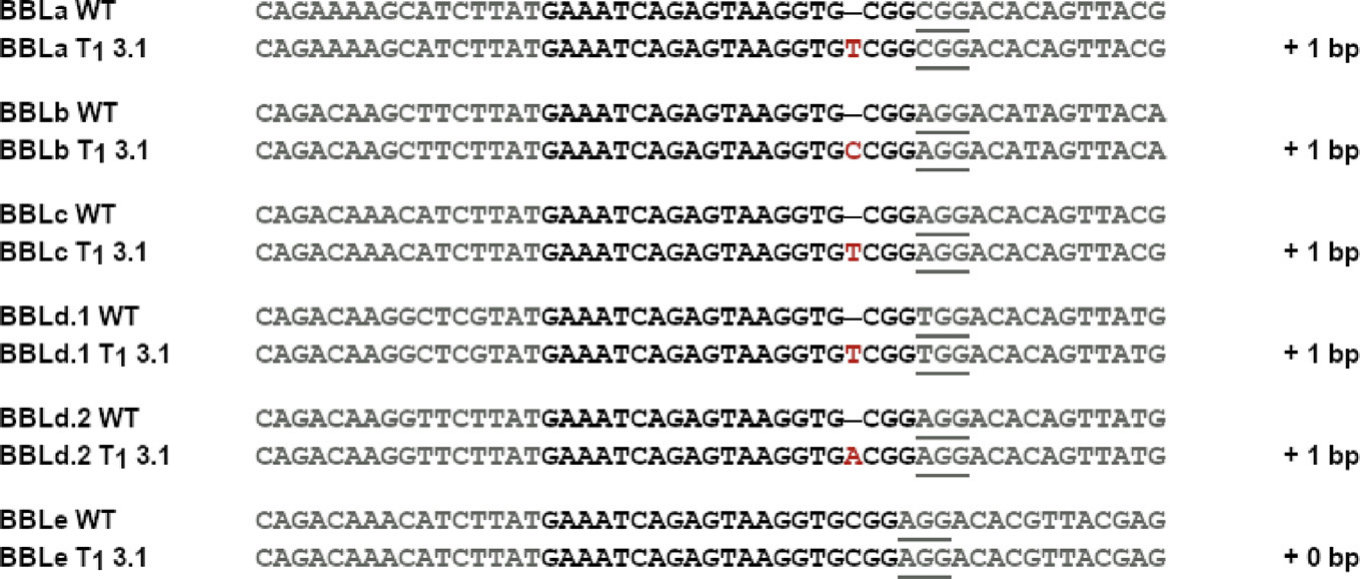
Analysis of plant T_1_ 3.1 on genomic level: Fragments of all six *BBL* genes (*BBLa* – *BBLe*) of the wild type (WT) and plant T_1_ 3.1 were amplified and cloned into a vector by Gibson Assembly for sequencing. Inserted bases are highlighted in red leading to a frame shift; PAM sequences are underlined and the gRNA sequence is shown in bold.

**Fig. 4:**
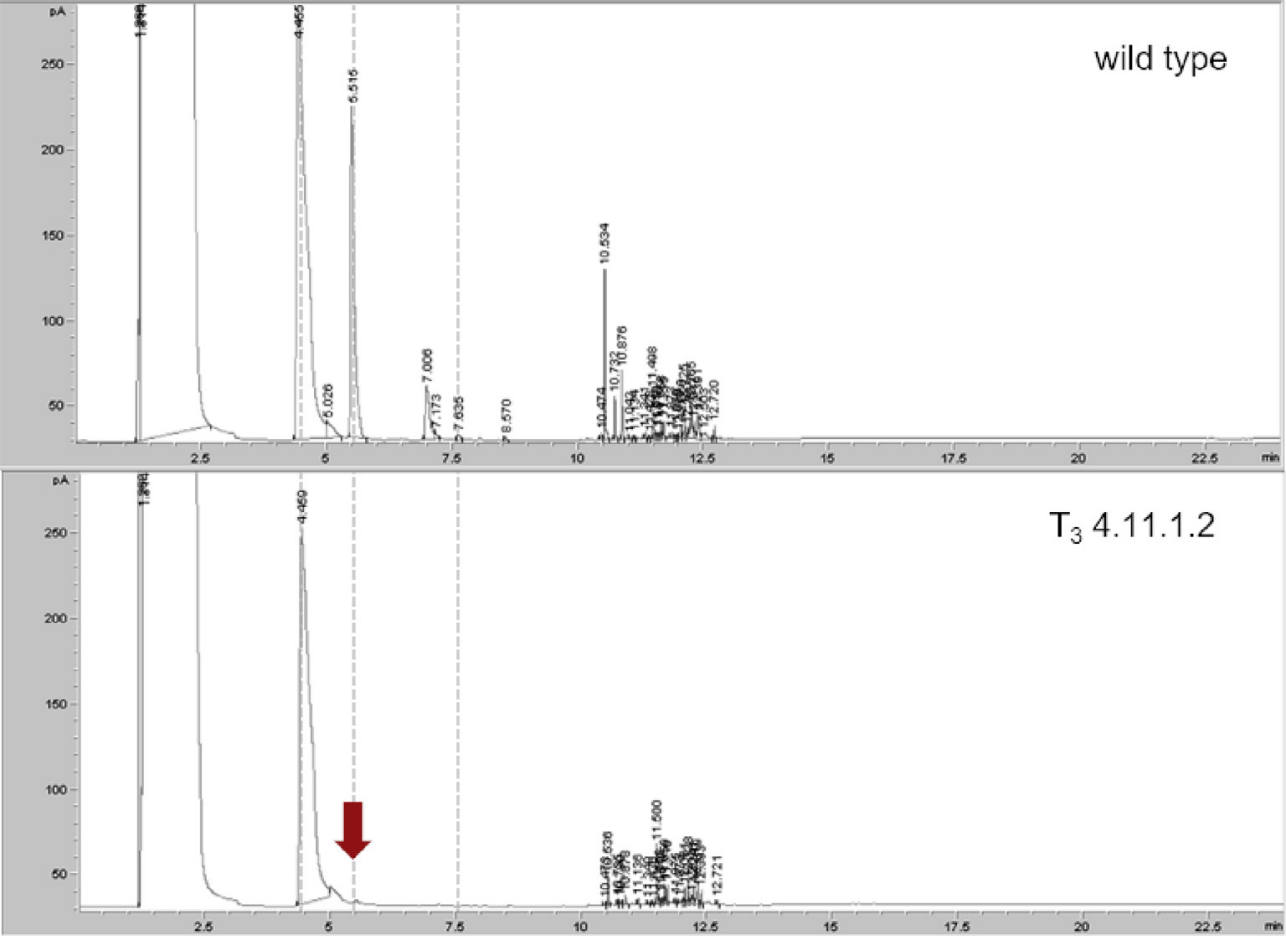
GC chromatogram of crude extracts of *Nicotiana tabacum* leaves: comparison of crude extracts of tobacco leaves of the wild type and the nicotine-free plant (T_3_ 4.11.1.2). The red arrow marks the retention time of nicotine. (1.8 min - injection peak; 4.4 min - internal standard (quinoline); 5.5 min – nicotine; 7.6 min – anatabine).

**Fig. 5:**
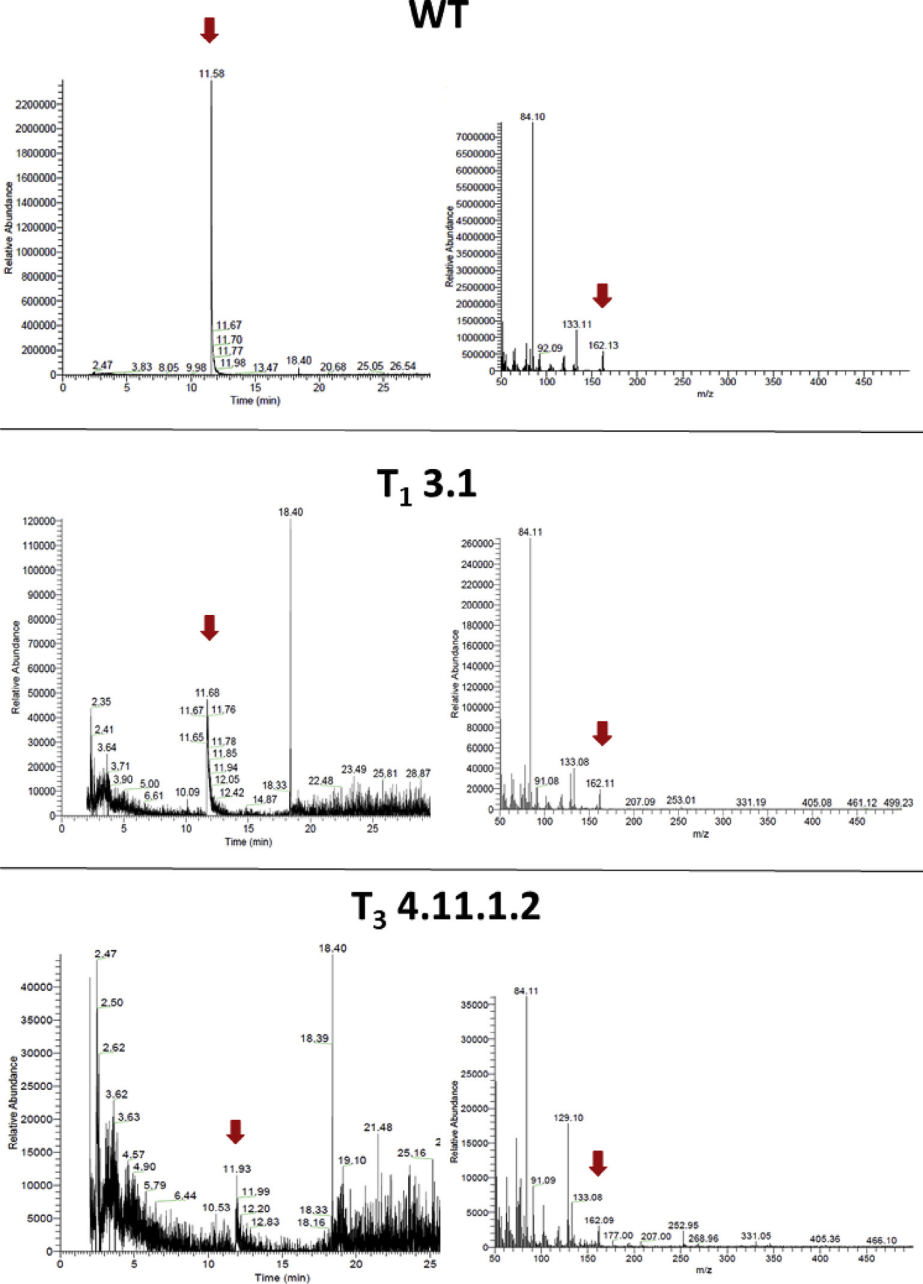
GC-MS analysis of plant extracts regarding the mass of nicotine: Extracts of the wild type (WT), plant T_1_ 3.1 and T_3_ 4.11.1.2 were analyzed with GC-MS measurements by the analysis of peaks corresponding to the *m*/*z* of nicotine (162.23 g/mol). Extracted Ion chromatogram (EIC) of the mass of nicotine and the corresponding mass spectra are shown.

**Fig. 6:**
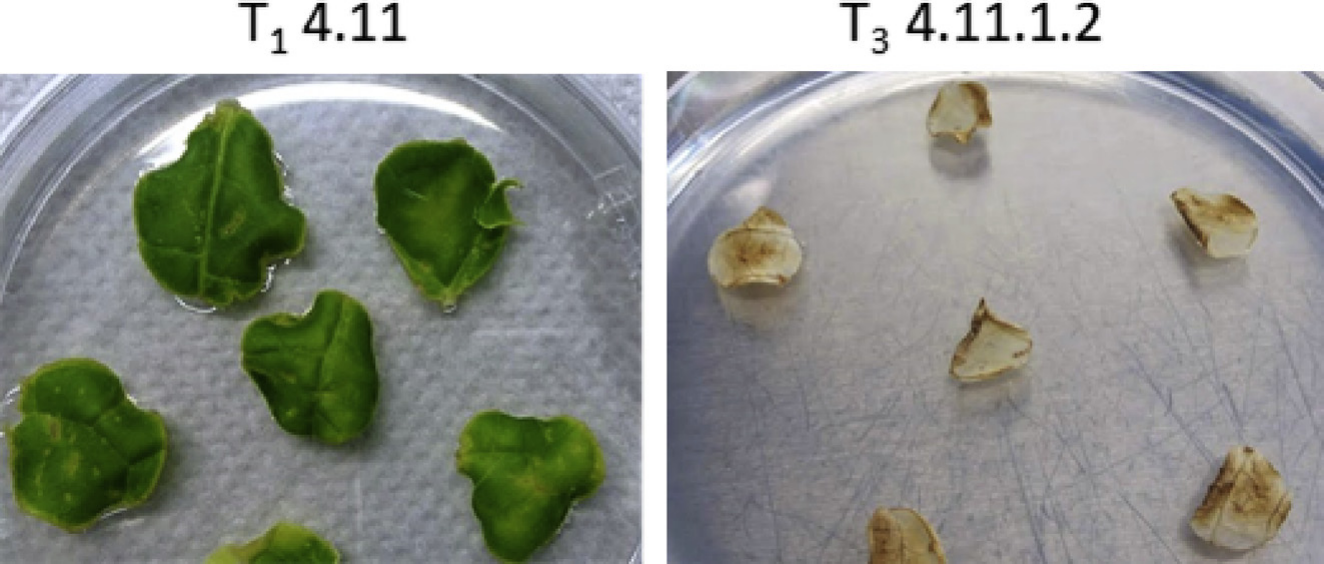
Test for non-transgenity of the nicotine-free plant: Leaf discs of the plants T_1_ 4.11 and T_3_ 4.11.1.2 were incubated in MS-medium with 6 mg L^−1^ PPT to test for the presence of the transformation cassette including the selection marker.

**Fig. 7:**
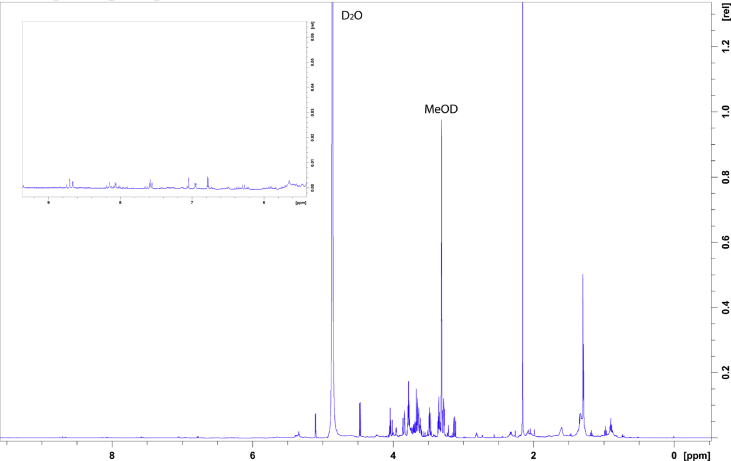
^1^H-NMR spectrum of wild type leaf-extract in MeOD: 20 mg leaf material of wild type leaves were freeze-dried and extracted in MeOD. The region of aromatics (6 ppm–9 ppm) is highlighted in the zoom-in insert, showing signals corresponding to nicotine standard (see Fig. 9).

**Fig. 8:**
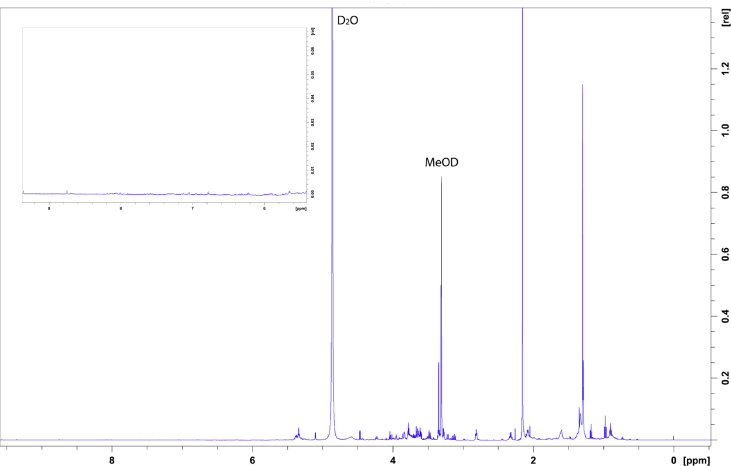
^1^H-NMR spectrum of nicotine-free plant leaf-extract in MeOD: 20 mg leaf material of nicotine-free plant leaves were freeze-dried and extracted in MeOD. The region of aromatics (6 ppm–9 ppm) is highlighted in the zoom-in insert, indicating the absence of nicotine.

**Fig. 9:**
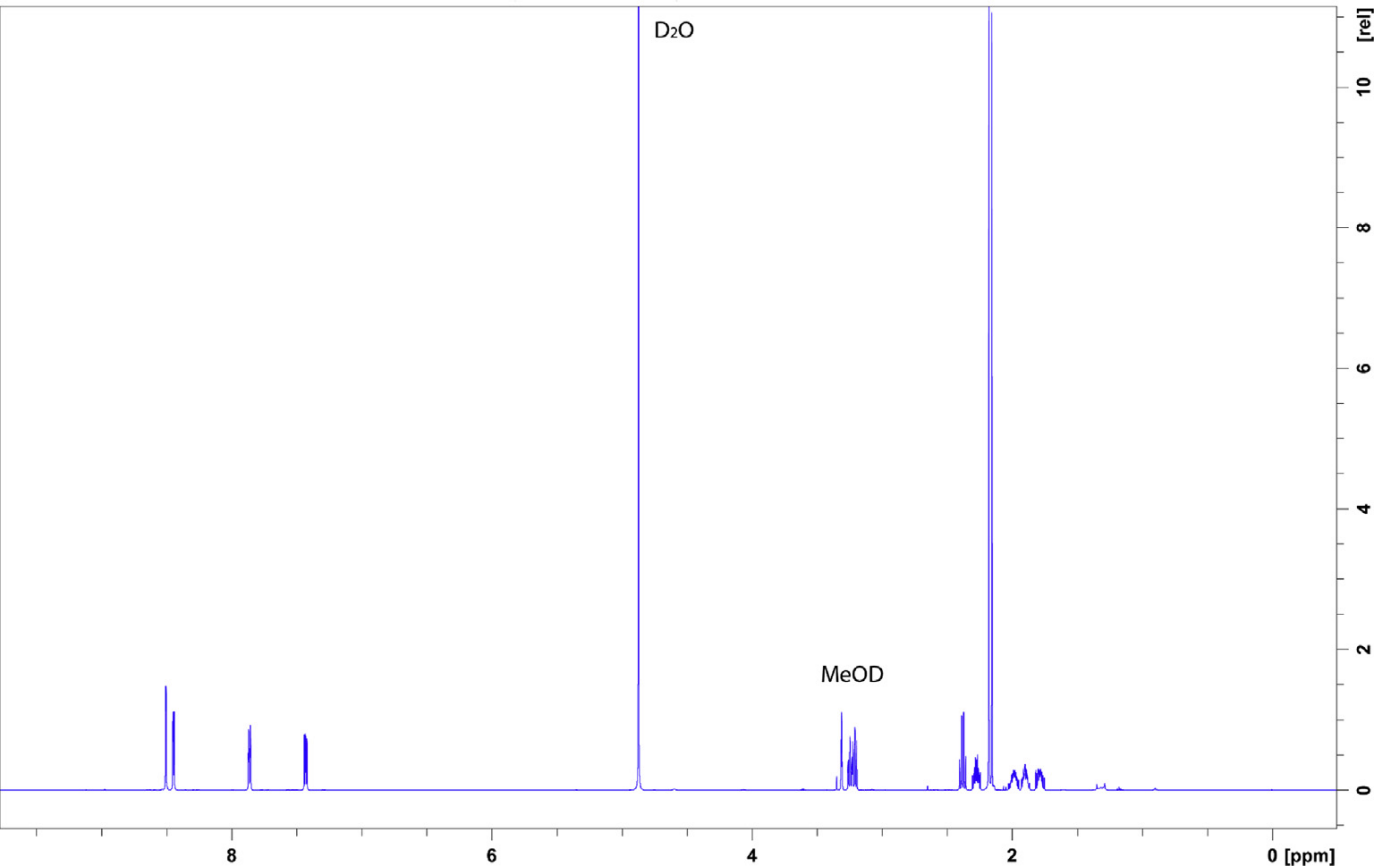
^1^H-NMR spectrum of nicotine in MeOD.

The data shows the generation of a nicotine-free and nontransgenic tobacco plant by CRISPR-Cas9 mediated gene editing. After regeneration of plants and testing them for transgenity (Fig. 1) the nicotine content was analyzed by GC measurements in wild type, T_0_ and T_1_ generations (Fig. 2). Genomic analysis of T_1_ 3.1 plant revealed that not all *BBL* (berberine bridge enzyme-like) loci were knocked out (*BBLe*)(Fig. 3). Therefore, further generations were grown and analyzed. The nicotine level of T_3_ 4.11.1.2 plant was reduced to 0.04 mg g^−1^ DW [1] (Fig. 4). This was additionally confirmed by GC-MS measurements (Fig. 5). The genomic analysis showed knockout of all *BBL*-loci [1]. The loss of the T-DNA cassette was proven for plant T_3_ 4.11.1.2 (Fig. 6). Finally, ^1^H NMR analysis showed no significant changes in primary metabolism (Fig. 7, Fig. 8, Fig. 9).

## 2 Experimental design, materials, and methods

### 2.1 Plant material

*Nicotiana tabacum* l. plants of the variety “Virginia Smoking Tobacco” (Strictly Medicinal Seeds LLC, United States) were cultivated on hydro culture at 25 °C under long day conditions (18 h light/6 h dark) with a light intensity of 110 μM m^−2^ s^−1^. For germination, seeds were surface sterilized with a sodium hypochlorite solution (1% active chlorine) and a few drops of Tween 20 for 10 minutes and washed three times with water before they were plated out on Murashige and Skoog Medium (4.4 g L^−1^ Murashige and Skoog Medium with Gamborg's Vitamins, 30 g L^−1^ sucrose, pH 5.8).

### 2.2 Plasmid construction

For the delivery of the CRISPR cassette to *N. tabacum* plants with *A. tumefaciens* a binary vector system was used [2]. The chosen sgRNA target sequence (GAAATCAGAGTAAGGTGCGG) for the *BBL* genes was cloned into the vector pChimera according to the author's instructions and the resulting vector was named pChimera-BBL. The gRNA chimera was subsequently cloned into the vector pCas9-TPC according to the author's instructions resulting in the vector pCas9-BBL, which was used for transformation experiments. All cloned vectors were verified by sequencing.

For the verification of the targeted mutagenesis on genomic level, gene sequences of the six *BBL* genes that include the target site were amplified with specific primers for each gene from genomic DNA of wildtype and transgenic plants. For sequencing, the gene fragments were either cloned into the vector pDionysos [3] by using the Gibson Assembly method or the PCR product was directly sequenced. Used primers are listed in Table 1.

**Table 1:** Primers used in the study.

Primer Name	Sequence (5′ → 3′)	purpose
BBL_gRNA_fwd	ATTGGAAATCAGAGTAAGGTGCGG	sgRNA target
BBL_gRNA_rev	AAACCCGCACCTTACTCTGATTTC	sgRNA target
SS129	CACAGGAAACAGCTATGAC	Colony PCR pChimera-BBL
SS42	TCCCAGGATTAGAATGATTAGG	Colony PCR; Sequencing pChimera-BBL
SS43	CGACTAAGGGTTTCTTATATGC	Colony PCR;
SS61	GAGCTCCAGGCCTCCCAGCTTTCG	Sequencing pCas9-BBL
BBLa_Gib_fwd	TCACACTGGCGGCCGCTCGAGCATGCATACTGCTACTGGAGCTGTTAC	Gibson Assembly pDionysos
BBLa_Gib_rev	ATAACTAATTACATGATGCGGCCCTTGCAGGTCTCAGCAGTACTC	Gibson Assembly pDionysos
BBLb_Gib_fwd	TCACACTGGCGGCCGCTCGAGCATGCATCTCTGCTACTGCAACTAGTGGA	Gibson Assembly pDionysos
BBLb_Gib_rev	ATAACTAATTACATGATGCGGCCCTATTTCCTCCTCCGCCACCTC	Gibson Assembly pDionysos
BBLc_Gib_fwd	TCACACTGGCGGCCGCTCGAGCATGCATTGGAGCAGGAGAAGGAGT	Gibson Assembly pDionysos
BBLc_Gib_rev	ATAACTAATTACATGATGCGGCCCTGGGCAACGTATTGTTTGGA	Gibson Assembly pDionysos
BBLd1_Gib_fwd	TCACACTGGCGGCCGCTCGAGCATGCATTTCGGTCTCTGCAACAAC	Gibson Assembly pDionysos
BBLd1_Gib_rev	ATAACTAATTACATGATGCGGCCCTGAAACTGGTCACGGTCTT	Gibson Assembly pDionysos
BBLd2_Gib_fwd	TCACACTGGCGGCCGCTCGAGCATGCATCTCTTCAGCGTTTGCTCATA	Gibson Assembly pDionysos, amplification
BBLd2_Gib_rev	ATAACTAATTACATGATGCGGCCCTCAAATCTACCGAAACATCATCT	Gibson Assembly pDionysos
BBLe_Gib_fwd	TCACACTGGCGGCCGCTCGAGCATGCATGGAGCAGGAGGAGTTACAAATC	Gibson Assembly pDionysos
BBLe_Gib_rev	ATAACTAATTACATGATGCGGCCCTTGGCGTCATCATTCTTAGCG	Gibson Assembly pDionysos
pDio_seq_fwd	CGGTTTGTATTACTTCTTATTC	Colony PCR
pDio_seq_rev	GATGTGGGGGGAGGGCGTGAATGTA	Colony PCR; sequencing
PCR_BBLa_fwd	ACTGCTACTGGAGCTGTTAC	amplification *BBLa*
BBLa_rev	TGCAGGTCTCAGCAGTACTC	amplification *BBLa*
BBLa_seq_fwd	GCACCTTTCATGCCGAAACC	sequencing *BBLa*
PCR_BBLb_fwd	TGGAGCAGGAGGTGGAGTTG	amplification *BBLb*
BBLb_rev	ATTTCCTCCTCCGCCACCTC	amplification *BBLb*
BBLb_seq_fwd	GCATCTTACATGCCGAAACC	sequencing *BBLb*
PCR_BBLc_fwd	TGGAGCAGGAGAAGGAGTTG	amplification *BBLc*
BBLc_rev	GGGCAACGTATTGTTTGGAG	amplification *BBLc*
BBLc_seq_fwd	GCATCTAACATGCCGAAACC	sequencing *BBLc*
PCR_BBLd1_fwd	TTCGGTCTCTGCAACAACTC	amplification *BBLd1*
BBLd1_rev	GAAACTGGTCACGGTCTTGG	amplification *BBLd1*
BBLd1_seq	ATTCGCAGCGTGCTCTAAAC	sequencing *BBLd2*
BBLd2_seq	CCGTCTCTGCTACAAATCTC	sequencing *BBLd2*

### 2.3 Plant transformation and regeneration

*A. tumefaciens GV3101::pMP90* cells were transformed with the plasmid pCas9-BBL as described previously [4]. Transformation of *N. tabacum* leaves and the followed plant regeneration were done according to an existing protocol with minor changes [5]. Plants were infiltrated with an OD_600 nm_ of 0.1 and incubated for 3 days at long day conditions. For plant regeneration infiltrated leaves were surface sterilized, cut into pieces and incubated on shooting medium (2.15 g L^−1^ Murashige and Skoog Medium with Gamborg's Vitamins, 30 g L^−1^ sucrose, 0.1 mg L^−1^ indole-3-butyric acid, 0.8 mg L^−1^ benzylaminopurine, 250 mg L^−1^ carbenicillin, 6 mg L^−1^ DL-phosphinothricin (PPT), 8 g L^−1^ agar, pH 5.2) under long day conditions. Developed shoots were transferred to rooting medium (2.15 g L^−1^ Murashige and Skoog Medium with Gamborg's Vitamins, 30 g L^−1^ Sucrose, 0.5 mg L^−1^ indole-3-butyric acid, 250 mg L^−1^ carbenicillin, 6 mg L^−1^ DL-phosphinothricin (PPT), 8 g L^−1^ agar, pH 5.2) for approximately 10 days.

### 2.4 Test for transgenic plants

Seeds from regenerated plants were collected and seeded out for growing the T_1_ generation. To test if these plants were transgenic, 7 days-old plantlets were sprayed with a solution of 100 mg L^−1^ PPT three times every two days. Survived plants were considered as transgenic and grown for further experiments.

Plants in T_2_ generation can be tested for the loss of the CRISPR-Cas9 cassette. For this purpose leaves of T_2_ generation plants were surface sterilized for 10 minutes with sodium hypochlorite (0.5% active chlorine) and a few drops of Tween 20 followed by three washing steps with water. Leaf discs were cut out with a cork borer and incubated on selection medium (4.4 g L^−1^ Murashige and Skoog Medium with Gamborg's Vitamins, 30 g L^−1^ sucrose, 6 mg L^−1^ PPT; pH 5.8) under standard growing conditions. Additionally, validation of the loss of the CRISPR cassette was done with PCR. Genomic DNA of the plants was isolated and amplification of the CRISPR cassette was done by the use of primers SS43 and SS61.

### 2.5 Plant extracts

For the extraction of alkaloids a modified version of the extraction protocol from Lewis et al. [6] was used. For the extraction 50 mg or 100 mg of ground leaves with 1 mL of a 2 N NaOH to moisten the sample in a glass vessel with a screw-cap. After 15 minutes of incubation time 5 mL of methyl tert-butyl ether (MTBE) containing 0.4 mg ml^−1^ quinoline used as an internal standard were added to the sample. Samples were incubated for 2.5 h with shaking at 200 rpm. For layer separation the glass vessels were stored without shaking overnight. The MTBE layer was used for gas chromatographic analysis.

### 2.6 Gas chromatographic analysis

Measurements of plant extracts were done with an Agilent Technologies 7890A GC system equipped with a flame ionization detector (FID) set to 300 °C and a VF-5ms column (CP8944; 30 m × 0.25 mm, ID 0.25 μm). H_2_ flow was set to 30 mL min^−1^, Air Flow to 400 ml min^−1^ and N_2_ flow to 30 mL min^−1^. Injector temperature was set to 250 °C and 1 μL of the sample was injected in splitless mode. Initial oven temperature was set to 110 °C, held for 1 minute and increased afterwards to 200 °C with a rate of 10 °C/min followed by an increase to 300 °C in steps of 25 °C/min. The temperature of 300 °C was held for 10 minutes.

For GC-MS measurements a Thermo Scientific Trace GC Ultra system with a ISQ mass spectrometer and a TG-SQC column (Thermo Scientific; 15 m × 0,25 mm, ID 0,25 μm) was used. Injector temperature was set to 90 °C and 1 μL of the sample was injected in splitless mode. Initial oven temperature was set to 60 °C for 1 min, followed by an increase of the temperature to 200 °C with a rate of 10 °C/min. Afterwards the temperature was increased to 300 °C with a rate of 25 °C/min which was held for 10 minutes. Helium was used as a carrier gas with a flow of 0.7 ml min^−1^.

### 2.7 ^1^H-NMR analysis

For NMR analysis, 20 mg of freeze-dried leaf material of the wild type and the nicotine-free plant or 10 mg of nicotine standard were mixed with 1 mL methanol-D4 and vortexed for one minute. After ultrasonication for 15 minutes the samples were centrifuged at 13,000 g for 5 minutes. Around 600 μL of the supernatant was filled into a 3 mm NMR-tube. ^1^H**-**NMR measurements were done at 25 °C and 600 MHz with the Bruker AV 600 Avance III HD (Cryoprobe) spectrometer. The data were analyzed using TopSpin 4.0.
